# A Small-Angle Neutron Scattering Environment for *In-Situ* Observation of Chemical Processes

**DOI:** 10.1038/s41598-018-24718-z

**Published:** 2018-05-08

**Authors:** Dominic W. Hayward, Leonardo Chiappisi, Sylvain Prévost, Ralf Schweins, Michael Gradzielski

**Affiliations:** 1 Stranski-Laboratorium fur Physikalische und Theoretische Chemie, Institut für Chemie, Technische Universität Berlin, Straße des 17. Juni 124, D-10623 Berlin, Germany; 2 Institut Laue-Langevin, 71 avenue des Martyrs, CS 20156, 38042 Grenoble cedex 9, France

## Abstract

A new sample environment for the observation of ongoing chemical reactions is introduced for small-angle neutron scattering (SANS) experiments which enables structural changes to be followed continuously across a wide *Q*-range in response to changes in the chemical environment. The approach is demonstrated and validated by performing single and multiple potentiometric titrations on an aqueous anionic surfactant solution (oligo-oxyethylene alkylether carboxylic acid in D_2_O) with addition times varying from 1 s to 2 h. It is shown that the continuous flow set-up offers considerable advantages over classical ‘static’ measurements with regards to sample throughput, compositional precision and the ability to observe fast structural transitions. Finally, the capabilities and ongoing optimisation of the sample environment are discussed with reference to potential applications in the fields of biology, colloidal systems and complex soft matter.

## Introduction

The past two decades have seen steady progress in the development of complex soft-matter systems and structures^1,2^. Key to this development have been the breakthroughs and improvements in measurement and characterisation techniques enabling a progressively deeper understanding of the underlying processes and interactions as well as the, often intricate, nanostructures in which they result^3–5^. In order to keep pace with or even accelerate these advances, it is important that characterisation methods continue to improve and that techniques are developed which can provide high throughput, high quality data. One successful approach to this challenge has been through the use of *in-situ* measurements to observe, either continuously or at discrete intervals, an evolving system using a combination of characterisation techniques. In this way, a range of measurements can be performed simultaneously, enabling processes such as particle growth, spontaneous self-assembly or polymerisation to be followed in real time and allowing for rapid screening of experimental conditions^6–8^ or mapping of phase diagrams^9,10^. These techniques have become particularly prevalent in the field of synchrotron small-angle X-ray scattering (SAXS), where the X-ray flux is high and it is possible to characterise samples efficiently and completely over very short time-scales. This capability is crucial when following dynamic processes, where it is not possible to perform further measurements or characterisation on intermediate states *ex-situ*. Moreover, even in cases where one could accurately reproduce many intermediate sample states, achieving the same resolution in the independent variable (e.g. sample composition, reagent concentration or monomer conversion) as for an equivalent *in-situ* experiment would not be possible due to the limited experimental time. For these reasons, there are a plethora of high throughput, *in-situ* sample environments available, combining SAXS measurements with a wide variety of complementary techniques, including: Raman spectroscopy^11–13^, UV-Visible spectroscopy^12,14^, Fourier transform infra-red spectroscopy^15^, fluorescence spectroscopy^16^ and conductivity^17^, turbidity^12^ and pH^18,19^ measurements.

Although synchrotron SAXS sources enable the measurement of very small sample volumes (due to tightly focussed beams) with high time resolution (typical flux at the sample position is ~10^13^ photons s^-1^)^20^, there are a number of reasons why they may not always be appropriate for the characterisation of soft condensed matter or biological structures. Firstly, due to the similar X-ray scattering length densities (SLDs) of organic materials, the contrast between different constituents of multicomponent systems is often low and difficult to model to the required degree of precision. Secondly, such materials are often prone to radiation damage, whereby the X-rays irreversibly alter the structure or chemistry of the samples^21^. By conducting small-angle scattering experiments with neutrons, rather than X-rays, it is possible to circumvent these issues. Not only does the non-systematic relationship between neutron scattering lengths and atomic mass numbers give rise to superior contrast conditions in multicomponent soft matter systems (particularly when used in conjunction with contrast variation techniques^22^), neutrons also do not cause radiation damage, even to sensitive biological systems^23–25^. In general, the drawbacks of the small-angle neutron scattering (SANS) technique are often cited as the lower flux (and therefore longer acquisition times/lower time resolution) and larger beam size (i.e. greater sample volume)^24,26^ and, as a result, the development of sample environments for SANS instruments is much less advanced than for their SAXS equivalents. However, due to a confluence of two important factors, this paradigm may be about to shift. Firstly, recent years have seen the refurbishment of many SANS instruments, benefiting from larger and faster detectors and improved coating technology in the guides^27–29^, leading to higher effective flux. Furthermore, a number of high-flux sources have recently come on-line or are expected to do so with the next few years^30–32^. Secondly, the total number of neutron scattering facilities worldwide is in decline and the amount of experimental time at SANS instruments is expected to fall^33^. The challenge, therefore, is to exploit the improvements at the facility level and mitigate the effects of decreasing experimental time, by expanding the range of *in-situ* sample environments available on SANS instruments. This will, in turn, allow the biological and soft matter communities to explore complex phenomena that have hitherto remained inaccessible to small-angle scattering experiments.

There has already been some recent progress in the development of SANS sample environments, particularly in the field of microfluidics, where high-resolution, rapid prototyping of sample cells has been used in conjunction with high-flux beamlines to investigate the flow-response of lyotropic, lamellar phases^34^ and perform rapid and detailed contrast-matching experiments^35^. Elsewhere, a number of custom-made environments have shown excellent promise including: simultaneous SANS and dynamic light scattering (DLS) measurements^36,37^, simultaneous SANS and size-exclusion chromatography (SEC)^38^ and *in-situ* SANS and pH measurements^39^. However, in each case, the experiments were complicated to set up and have not seen widespread up-take. In this letter, a chemical reactor with a flow-through observation cell is presented which enables the effects of changes in chemical composition, e.g. due to ongoing chemical reactions, to be measured continuously. This approach not only increases the informational content of the experiment, via high-throughput and compositional precision, it also reduces the likelihood of data gaps arising whenever rapid transitions occur. In addition, the combination of high compositional precision and time-resolution allows for the exploration of non-equilibrium states that would go unnoticed in a classical ‘static’ approach. It will also enable systematic investigations to be conducted of unstable regions in phase diagrams, opening the door for a potential exploitation of non-equilibrium phenomena. In conjunction with the advantage of contrast variation unique to neutrons, the sample environment also offers new perspectives to the biology and soft matter communities to investigate multi-component systems as they evolve during chemical reactions or biochemical processes. The feasibility and capabilities of the set-up are demonstrated herein via an investigation of the morphological evolution that occurs in an aqueous surfactant system as a result of changes in pH and salt concentration.

## Results

**Figure 1:**
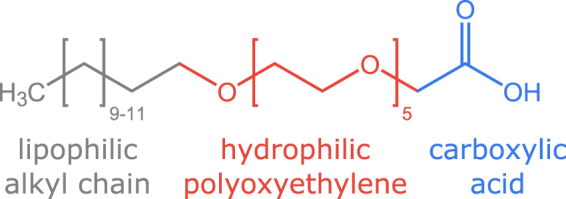
Chemical structure of the oligo-oxyethylene lauryl ether carboxylic acid surfactant (C_12-14_O-EO_5_CH_2_COOH) used in this experiment.

**Figure 2:**
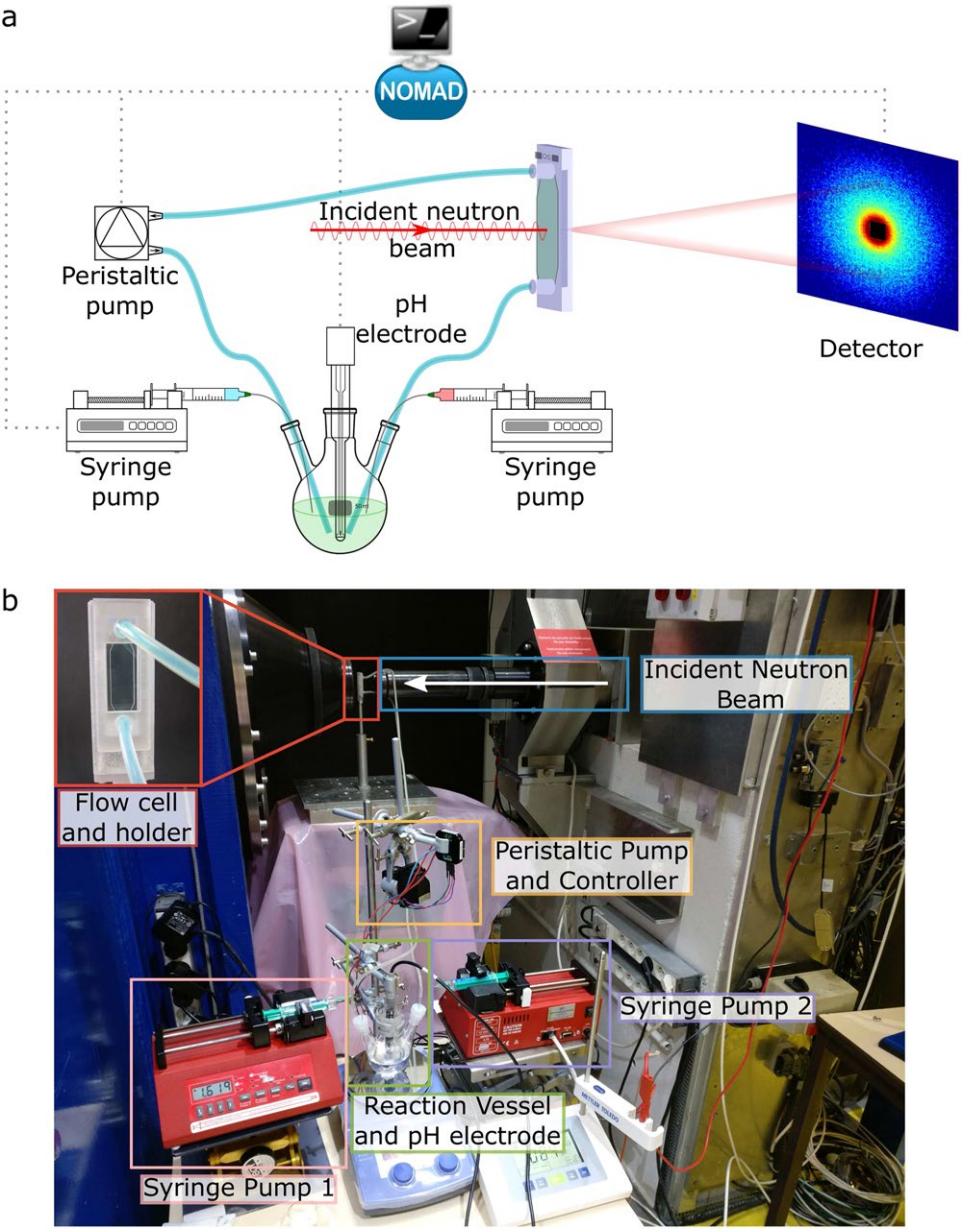
(**a**) Schematic of experimental set-up with dotted lines representing data connections. (**b**) Photograph of the experimental set-up on the D11 instrument. The NOMAD icons in (**a**) are used with permission from the Institut Laue-Langevin, the publisher and rights holder of the NOMAD instrument control software.

The sample environment was characterised using aqueous solutions of polyoxyethylene alkylether carboxylic acids (AECs), a class of surfactants with strongly pH-responsive properties^40–42^. AECs are made up of three distinct components, a hydrophobic alkyl chain, a hydrophilic polyoxyethylene block and a terminal hydrophilic carboxyl group. Although superficially similar to non-ionic polyoxyethylene alkylethers, the presence of an ionisable component imparts rather different properties and self-assembly phase behaviour to AECs compared to their non-ionic analogues^43,44^. In turn, these, often enhanced, properties have given rise to a wide range of applications from detergency^45^ and textile processing^46^ to enhanced oil recovery^47–49^. The complex, pH-dependent phase behaviour makes AECs an ideal candidate for the characterisation and commissioning of the continuous flow chemical reactor sample environment. Specifically, the AEC, oligo-oxyethylene(5) lauryl ether carboxylic acid, shown in Fig. 1, is known to undergo a dramatic structural transition between large vesicles and small micelles over a narrow pH range^40,42^, as the effective surface area of the head-group increases due to electrostatic repulsion. It was therefore selected for further study in this work. The experimental set-up, shown schematically and photographically in Fig. 2, comprised: a reaction vessel, a pH electrode, a peristaltic pump, an observation cell and two syringe pumps. SANS measurements were performed on the D11 instrument at the ILL neutron scattering facility in Grenoble with a wavelength of 6 Å and maximum flux of 10^8^ neutrons cm^-2^s^-1^ at the sample position^27^. The pD (= -log ${{\rm{C}}}_{{{\rm{D}}}_{{\rm{3}}}{\rm{O}}}$^+^) of the solutions was varied by addition of NaOD (0.1 M or 1 M in D_2_O) and DCl (1 M in D_2_O) to the solution, where NaOD and DCl are the deuterated analogues of NaOH and HCl respectively. Further experimental details are provided in the Methods section.

**Figure 3:**
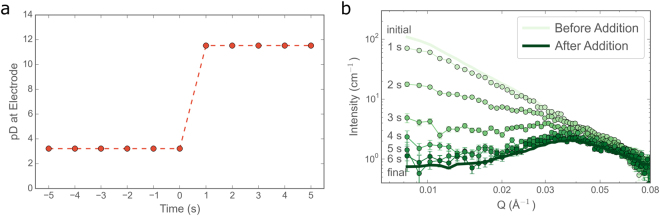
(**a**) Recorded pD values before and after fast addition of NaOD (1.2 mL 1 M NaOD added over 1.2 s, starting at t = 0 s), (**b**) azimuthally averaged scattering data showing the evolution of the nanostructure after addition of NaOD. Solid lines represent data averaged over the 30 s before addition and 30 s after the structure has equilibrated (i.e. 7-36 s after addition).

In order to characterise the time resolution of the continuous flow set-up, an initial ‘fast-addition’ experiment was performed, whereby 1.2 mL of 1 M NaOD were added to 45.5 mL aqueous surfactant solution over 1.2 s and data were acquired continuously with a frequency of 1 s^−1^. The pD measured at the electrode in the solution changed instantaneously on addition of NaOD (within the 1 s time resolution of the measurement, see Fig. 3a) whilst the form of the azimuthally-averaged scattering data evolved over the course of approximately 6 s, after which time no further changes were observed (see Fig. 3b). It can be readily seen, both from the size of the error bars as well as the well-defined form of the scattering curves, that a 1 s exposure is adequate to provide data of sufficient quality for further analysis, even for absolute scattering intensities close to 1 cm^-1^. The observed changes in the scattering curves are ascribed to homogenisation of the sample in the observation cell. This is considered likely for two reasons: firstly, the time taken for the surfactant solution to complete one circulation of the flow loop was determined to be ~5 s (details can be found in section S4 of the Supporting Information). Secondly, measurements of the response time for the vesicle-to-micelle transition following a pH-jump, in a similar system containing decanoic acid/decanoate, showed structural rearrangements taking place within 50 ms^50^.

**Figure 4:**
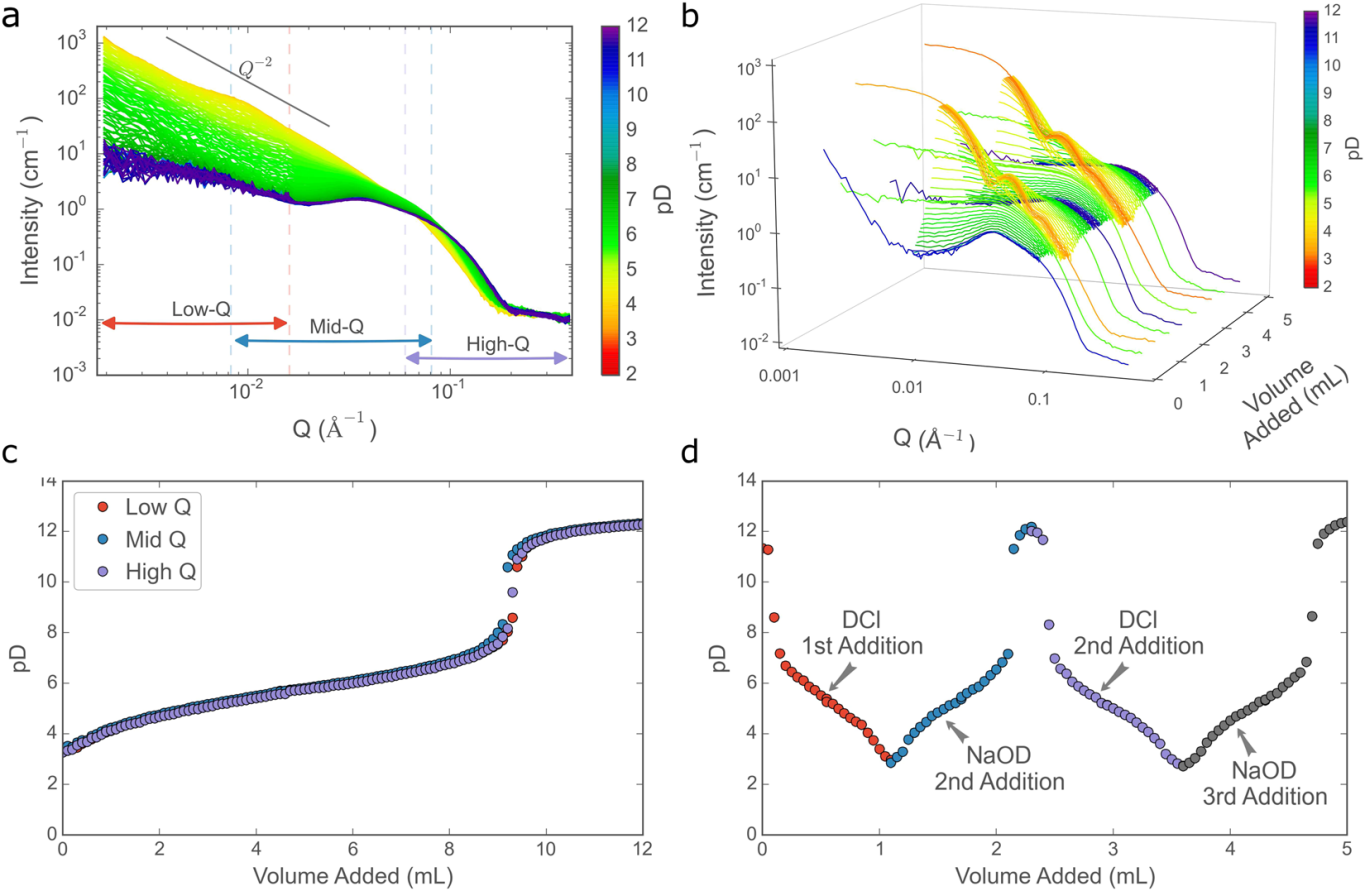
Azimuthally averaged scattering data showing: (**a**) the structural evolution occurring on slow addition of dilute NaOD and (**b**) the structural changes that occur with subsequent successive additions of DCl and NaOD (with a consequent gradual increase in the concentration of NaCl). The colour of the lines corresponds to the average pD at which the measurement was conducted. Plots (**c**) and (**d**) show the corresponding titration curves of: (**c**) the three repeated single titrations of 0.1 M NaOD, carried out at different detector distances and (**d**) the multiple successive titrations of 1 M DCl and NaOD.

Once the homogenisation time of the set-up had been determined, an experiment was undertaken to follow the structural evolution of the self-assembled surfactant aggregates at a high resolution with respect to the pD of the solution. This was done by changing the pD from an initial value of ~2.5 up to ~12 via the slow, continuous addition of 12 mL of 0.1 M NaOD over 2 hours, with an addition rate of 0.1 mL/min. The titration was performed three times, once at each sample-to-detector distance, in order to follow the structural evolution over a broad *Q*-range. The titration curves for the repeat measurements at different detector configurations are shown in Fig. 4c in order to establish the validity of combining scattering data from separate measurements. The resulting SANS curves from the three different experiments are shown in Fig. 4a, combined according to the volume of titrant added and rebinned to reduce the number of data points (details of the rebinning procedure are given in section S2 the Supporting Information). The overlap between the detector configurations is very good, further demonstrating the validity of this approach. Without making any prior assumptions as to the morphology of the self-assembled structures, observation of the scattering curves enables the identification of a number of noteworthy trends. At low pD, the surfactant molecules self-assemble into large, locally-flat structures (such as vesicles or bilayers) as evidenced by the *Q*^-2^ dependence. As the pD increases, the morphology gradually evolves to favour smaller, interacting structures, as shown by the drop in intensity at low-*Q* and the appearance of a distinct structure factor peak at 0.035 Å^-1^. These changes occur between pD 3 and pD 6, due to the ionisation of the surfactant head-group^42^. Thereafter, once the head-group is fully charged, the form of the scattering remains constant up to pD 12.

The azimuthally-averaged intensity was analysed according to a two-component model^42^ consisting of a population of non-interacting, unilamellar, core-shell vesicles and a population of oblate ellipsoidal core-shell micelles interacting via a charged hard sphere structure factor. In both cases, the hydrophobic component was considered to be anhydrous and the EO shell was assumed to be partially hydrated. A full description of the model can be found in section S3 of the Supporting Information. Nonlinear least-squares fits were performed by allowing the fraction of surfactant in micellar form, micellar radii, hard sphere radius, vesicle radius and vesicle polydispersity to refine. Fits were conducted on an absolute scale and the following constraints were imposed, based on the experimental conditions and geometric considerations concerning the surfactant molecule. The volume fraction of dispersed material was fixed to the calculated value based on the volumes of: surfactant, D_2_O, acid and base in the reaction vessel for the given measurement, the shell thickness of the micelles could not exceed the length of a fully extended EO block (16 Å)^51,52^ and the semi-minor axis of the micelle cores could not exceed the length of a fully extended hydrophobic block (16 Å)^53^. The total thickness of the vesicle shells was fixed at 29 Å in accordance with previous work^42^. In addition, to ascertain the effects of continuous flow on the self-assembled structures, SANS measurements were performed on the surfactant solution under both static and flow conditions. Although some alignment of the anisotropic structures was observed at low pD, the azimuthally-averaged scattering data showed no significant differences between static and flow conditions (Figure S2) and shear effects are therefore not considered in the following discussion of the results. The results are described in section S4 of the Supporting Information and the advantages and disadvantages of measuring samples under flow conditions are discussed in more detail in the discussion section.

**Figure 5:**
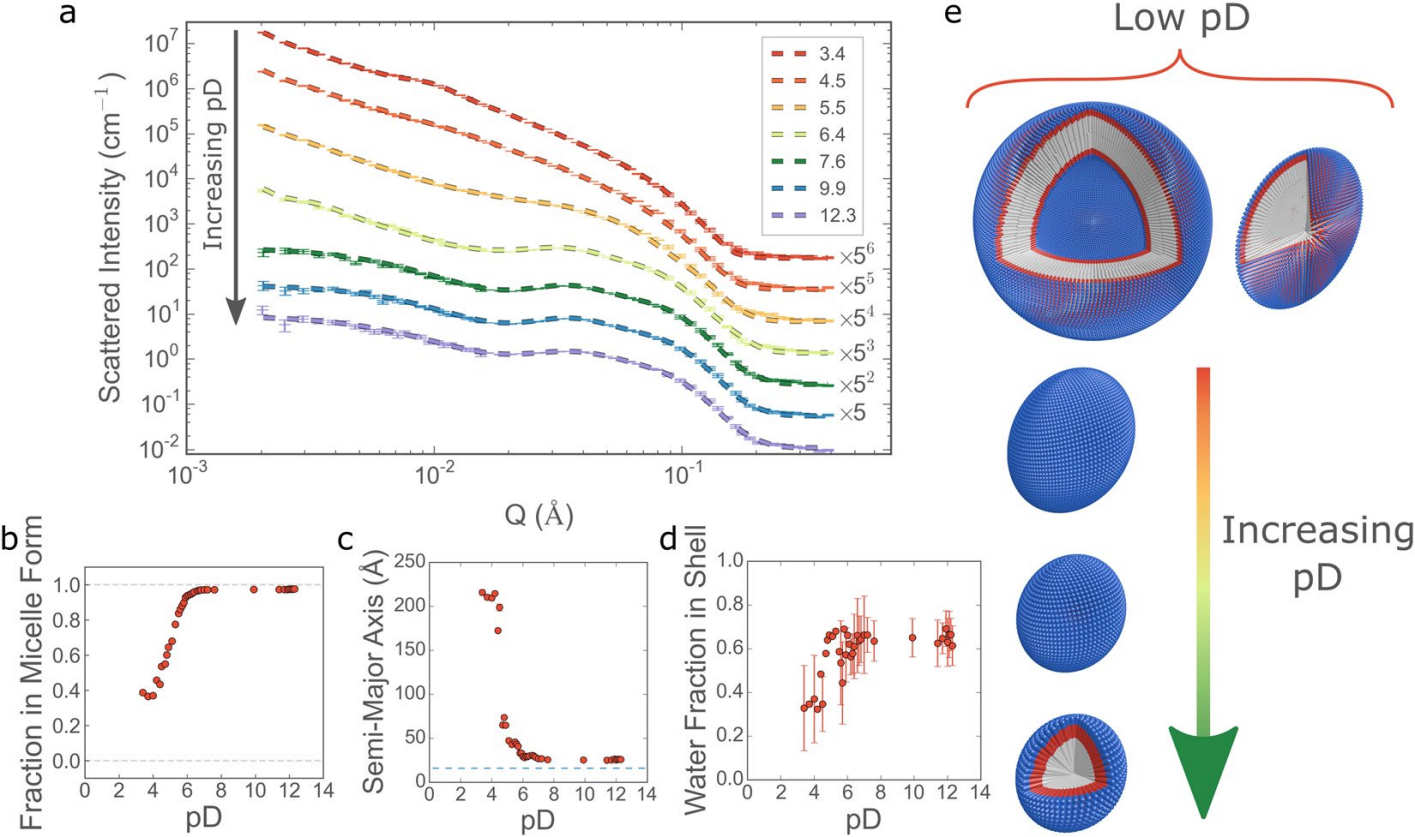
(**a**) Representative azimuthally-averaged intensity profiles vs *Q* from the single NaOD titration. Data are represented by thin horizontal lines with error bars and fits to the model described in the text are shown as thick dashed lines. pD values for each dataset are given in the legend and all profiles with an adjacent number have been shifted in intensity for clarity. Plots (**b**–**d**) show the evolution of the fitted parameters with pD: (**b**) fraction of surfactant in micellar form, (**c**) semi-major axis of the ellipsoidal micelles (the maximum semi-minor axis is displayed as a dashed line for reference) and (**d**) volume fraction of D_2_O in the micelle shell. A cartoon of the corresponding structures, as they are interpreted in this work, is shown in (**e**) (not to scale).

From Fig. 5a, showing representative fit curves, it can be seen that the model is able to capture the evolution of the scattering patterns with a high fidelity. Coupled with the continuous nature of the measurement and the consequent high volume of scattering data, this allowed for the comparison of experimentally determined structural parameters such as the size, fraction of surfactant in micellar form and water content of the aggregates to be followed as a function of the experimental variable, pD. The results are shown in Fig. 5b–d. At low pD, prior to the addition of NaOD, the surfactant self-assembles into large, polydisperse, locally-flat structures, as indicated by the extended *Q*^-2^ scattering power law as shown in Fig. 4a and as has been reported previously^54^. These structures are interpreted in the model as a mixture of large vesicles and ellipsoidal micelles. Although the overall size and polydispersity of the vesicles cannot be properly determined as they fall outside of the probed *Q*-range (i.e. >2500 Å), and the presence of extended bilayers cannot be ruled out, the vesicle interpretation was considered more probable, given the appearance of smaller vesicles at high pD and in the presence of an inert salt (*vide infra*). The volume fraction of ellipsoidal micelles with respect to vesicles is given in Fig. 5b, it confirms and quantifies the rapid transition from vesicles to micelles in the pD range 4–6. This pD range also sees a dramatic change in the shape of the ellipsoidal micelles with the semi-major axis (i.e. the larger of the two cross-sectional radii) decreasing from ~200 Å to 30 Å, as can be observed in Fig. 5c. The geometry of an ellipsoid with a 15:1 ratio between major and minor axes is shown schematically in 5e. These quasi disc-like objects would account for both the extended *Q*^−2^ dependence at low pD, as well as the observed alignment behaviour under flow. Above pD values of ~6, the self-assembled structures appear to be almost exclusively (>97%) micellar, where the form of the micelles is much closer to the ‘traditional’ spherical conception of surfactant-based micelles. The volume fraction of D_2_O in the micelle shell, as calculated from the fitted parameters, increases with increasing pD (shown in 5d). In this case, the variability between points is considerably higher than for the geometric parameters, however the general trend is clear; significant hydration of the EO block in the shell occurs as the pD increases from 4 to 6. This increase in hydration is a direct consequence of the increasing electrostatic repulsion between the carboxylic headgroups as the degree of ionisation increases.

**Figure 6:**
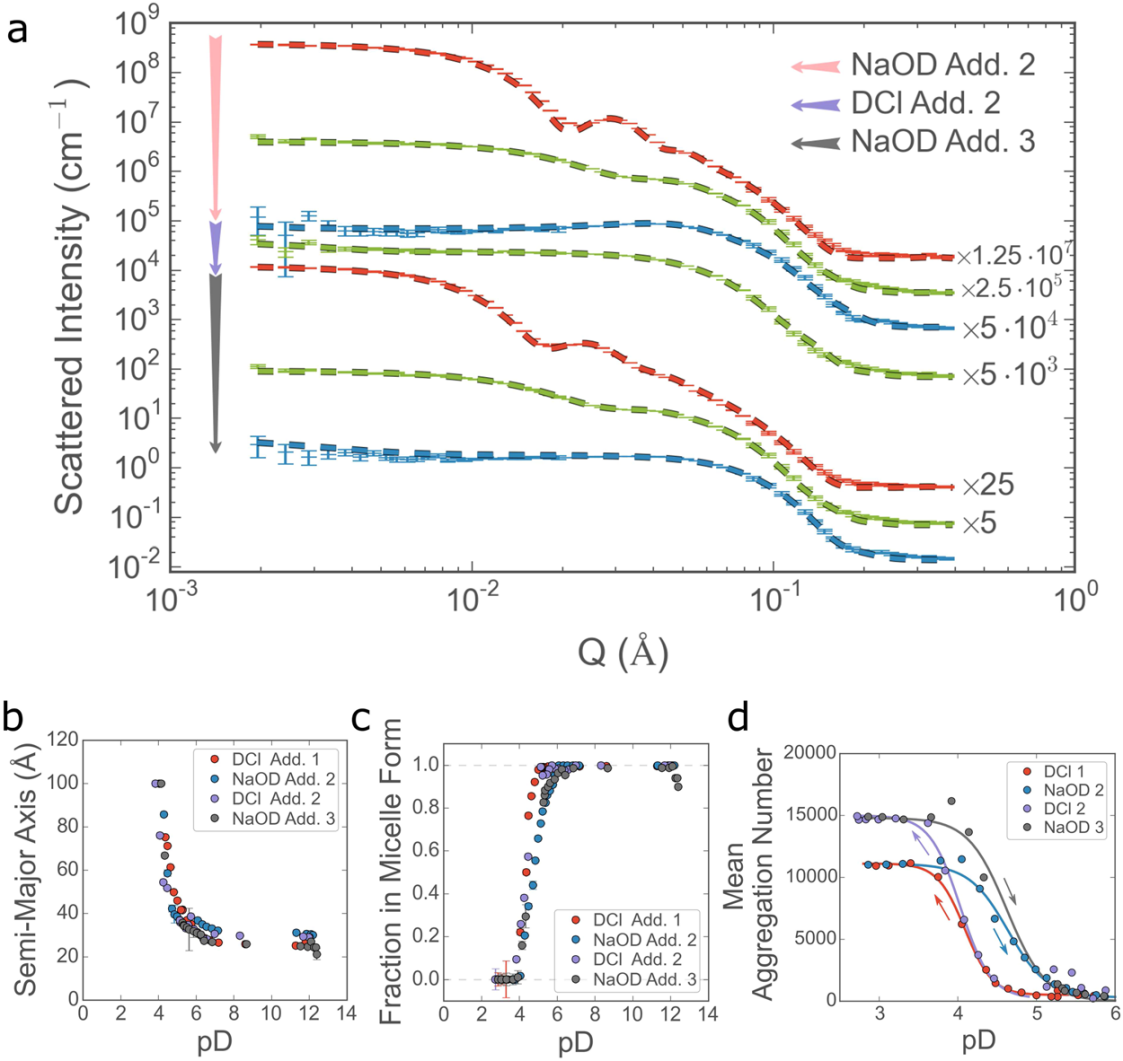
(**a**) Representative azimuthally averaged intensity profiles vs *Q* from the multiple titrations of DCl and NaOD. The dashed lines are fits of the model described in the text. All profiles with an adjacent number have been shifted in intensity for clarity. The colours correspond to the similar pD values: red ≈ 2.9, green ≈ 4.9, blue ≈ 11.7. Data are represented by thin horizontal lines with error bars and fits are shown as thick dashed lines. Plots (**b**–**d**) show the evolution of the fitted parameters with pD: (**b**) semi-major axis of the ellipsoidal micelles, (**c**) volume fraction of micelles and (**d**) mean aggregation number of the self-assembled aggregates at low pD. The solid lines and arrows in (d) are given as a guide for the eyes.

A final investigation was conducted with a view to simulating more complex experimental conditions, where multiple components or reagents are added at predetermined times during the reaction, or where the reversibility of a process is to be investigated. Within the context of a titration experiment, the additional complexity was achieved by observation of the scattering over multiple successive potentiometric titrations of NaOD and DCl. This experimental protocol gives rise to a progressively increasing NaCl concentration in the surfactant solution which increases to 24 mM after the first DCl addition and 48 mM after second addition (see the Methods section for further details). To make most efficient use of the available time, the scattering was measured continuously during the additions at a single detector configuration (mid-*Q*), with pauses between and half-way through each addition to measure the full *Q*-range. In this way it was possible to both observe the structural evolution in continuous fashion over the most relevant size regime and also have regular access to the complete scattering picture. The scattering and titration data are given in Fig. 4b and d respectively. Comparing the fitted parameters in Figs 5 and 6, a number of effects arising from the increasing salt concentration can be immediately identified. Figure 6b shows that the shape of the ellipsoidal micelles does not appear to be strongly influenced by the presence of NaCl. The largest dimensions, present in Fig. 5c at low pD, do not appear, as the fraction of surfactant in micellar form drops to zero. This can be seen in Fig. 6c, which shows that at pD < 4, the surfactant is entirely in vesicle form and at pD > 6 the surfactant is entirely micellar (in contrast to the salt-free solution where both components are present at all pD values). Furthermore, the presence of an inert salt has a pronounced effect on the radius of the vesicles formed at low pD. The plateau at low-*Q* and the oscillations in the scattered intensity reveal that the vesicles are both an order of magnitude smaller (from >2500 Å to ~120 Å) and much less polydisperse in the presence of salt, this is also reflected in the fitted parameters shown in Figure S4 in the Supporting Information. This effect has since been observed in other AEC systems^55^. It is also worth noting that radii of 109 to 128 Å are uncommonly low and such small vesicles are only rarely seen, for example when induced by the presence of a cosurfactant in ionic surfactant systems^56^. Finally, it can be seen that the overall aggregate size depends not only on the pD of the solution and the salt concentration but also on the ‘direction of travel’. On addition of DCl, the mean aggregation number begins to increase, due to the formation of vesicles, at pD 5, reaching a maximum at pD 3.5. On subsequent addition of NaOD however, the vesicles appear to persist up to pD 4.5, and the smaller aggregation number, corresponding to the surfactant in micellar form, is regained at pD 6.0. It is thought that the reasons behind this hysteresis behaviour may be due to the slower kinetics exhibited by the vesicles as compared to the micelles. This disparity is expected and can be ascribed to the much larger aggregation number in the case of the vesicles. Crucially, although the results in Fig. 6b and c could, in principle, have been found using conventional static measurements (although in practice this would not be possible as experimental time at large scale facilities is limited), the results in Fig. 6d could only be observed using a continuous flow sample environment.

The purpose of highlighting the aforementioned features in the scattering data is primarily to demonstrate the type of detailed information that can be extracted from *in-situ* scattering experiments of complex, evolving systems. It must be noted that describing systems which contain structures of different shape and size, a common occurrence in evolving systems, generally requires a large number of free parameters. Although this will introduce a certain degree of ambiguity in the results (due to correlations between dimension parameters of similar size for example), these ambiguities can be mitigated by the addition of information gained from other sources or techniques. In this case, the maximum dimensions of the hydrophobic and hydrophilic components of the molecule are known and can be constrained within the fit. As a result, the fits are robust and the trends are clear. In summary, as long as the limitations are not ignored, it is possible to extract a large amount information from *in-situ* scattering experiments, despite their apparent complexity.

## Discussion

In this work, a continuous-flow chemical reactor sample environment has been demonstrated and characterised via a potentiometric titration experiment. The set-up permits complex reactions with multiple reagents to be monitored with a time-resolution down to 6 s. Critically, this allows chemical reactions to be monitored in real time, providing insights into mesoscopic structural changes and non-equilibrium structures that may occur as the system evolves. It is quick and straightforward to assemble and enables the efficient collection of large amounts of data with respect to equivalent experiments conducted via discrete reaction sampling which would require both more time and a larger sample volume. The sample environment can be controlled entirely from the SANS instrument control platform and has been designed to be as easy to use and robust as possible so that it can be made available to all interested users with successful beam-time applications.

As was touched upon in the results section, use of the continuous flow set-up detailed in this work, necessarily entails observing samples under shear. The existence of shear forces acting on the sample along the flow-loop and in the quartz sample cell presents both advantages and disadvantages when devising experimental protocols. On the one hand, the application of a shear field (as occurs during simple stirring for example) is known to cause changes in the morphology of certain soft matter systems^57–62^ and may introduce additional complexity to subsequent data analysis. On the other hand, shear forces can be used to align anisotropic particles, whereupon preferred orientation can be exploited to gain additional information on the size, form, and symmetry of the structures under investigation^63–67^. If anisotropy is present, it can be evaluated straightforwardly, even across very large datasets, using software such as *SASET*^68^ (freely available on request), to provide an insight into how the anisotropy evolves during an experiment. An example of such an analysis is demonstrated in Figure S3. Crucially however, as the flow-rate of the peristaltic pump can be continuously varied over a wide range (0–90 mL/min), the wall shear rate in the sample cell (τ_*w*_~0-920 s^−1^) can be adapted to suit the requirements of each individual experiment (c.f. wall shear rates in human blood vessels^69,70^ reach up to 1500 s^−1^).

**Figure 7:**
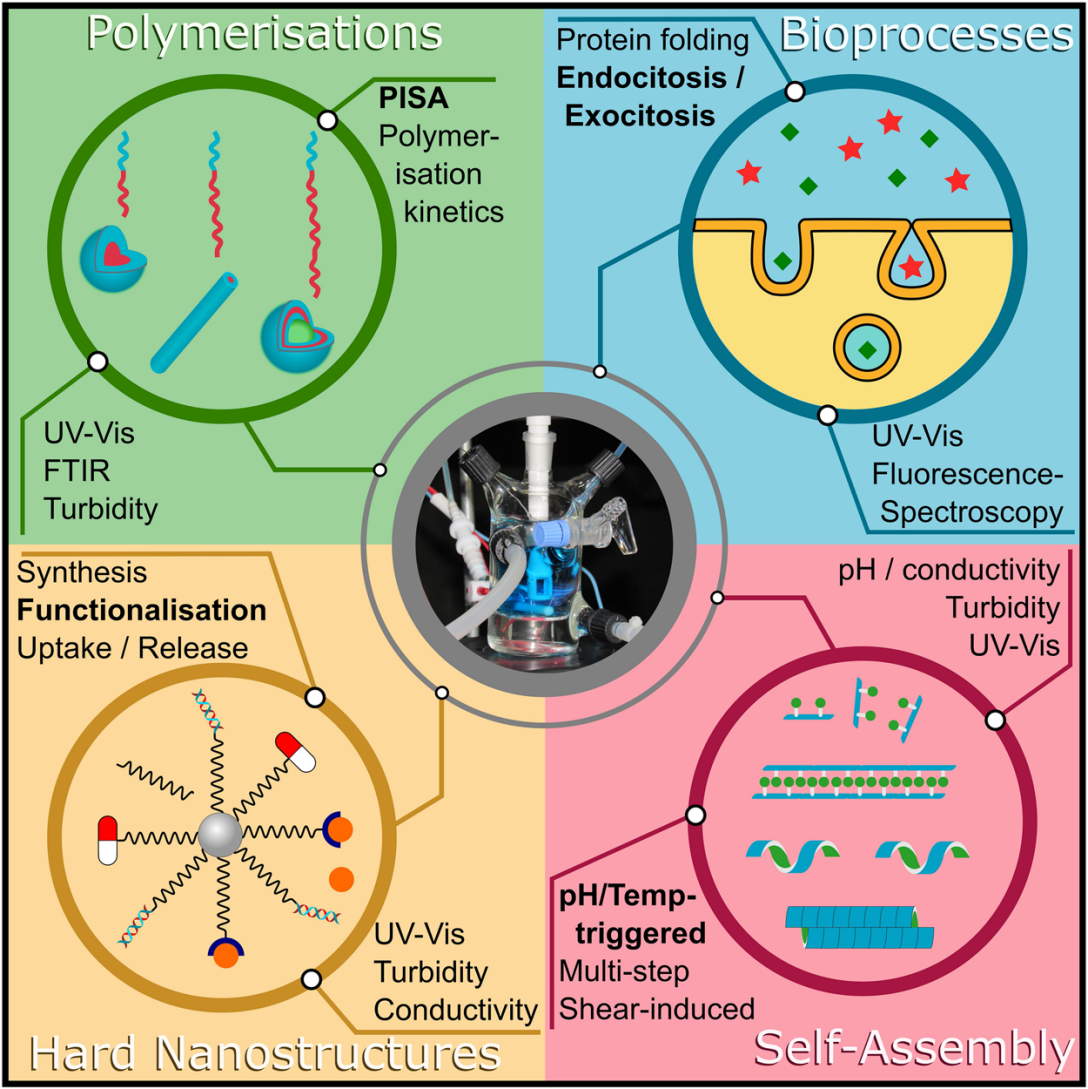
Illustrations of potential applications for an *in-situ* continuous-flow sample environment. The central photograph shows a prototype small-volume (25 mL), sealable, jacketed reaction vessel with turbidity probe, in operation on the D11 beamline.

Work is ongoing to shorten the flow-loop and reduce the sample volume from ~50 mL to ~5 mL. A 25 mL sealable, jacketed reactor vessel (pictured in Fig. 7) has already been tested successfully and a 5 mL version is currently under commissioning. This will extend the range of potential applications to cover systems where large sample volumes are unavailable or impractical. It will also reduce the homogenisation time down to the order of 1 s, making the continuous flow technique complementary to existing stopped-flow type methods with the advantages of a higher number of possible reagents (limited only by the number of syringe pumps) and the ability to perform additions at any point during the measurement (i.e. during course of the reaction). In conjunction with the temperature control capability included in the forthcoming iteration, this will allow for experiments of almost limitless complexity. It is also envisaged that additional complementary characterisation techniques will be included within the flow loop. Aside from pH electrodes, these will include: conductivity and turbidity probes, refractometry, viscometry, densitometry, UV-Vis absorption spectroscopy and Fourier transform infra-red spectroscopy (FTIR). The exact configuration of auxiliary instruments can then be chosen based on the sample system under investigation and the desired experimental outcomes.

In principle, there are three criteria that determine whether an *in-situ* continuous-flow SANS set-up is required for a given experimental system; the presence of one or more organic components, structures (or features) in the 1–100 nanometre size range and, crucially, structural transitions that occur in response to compositional alterations. Although these criteria do not explicitly rule out the possibility of performing analogous ‘static’ measurements, whereby every composition is prepared and measured individually, in most cases it would be impossible to match the time-efficiency and compositional resolution provided by the equivalent continuous-flow measurement. These criteria encompass a wide range of possible experiments across many scientific disciplines from vesicular transport in cells^71^ through polymerisation-induced self-assembly (PISA)^72^ and nanoparticle theranostic applications^73^ to pH triggered self-assembly of biomolecules^74^. Examples of possible experimental systems that could benefit from *in-situ* SANS measurements, alongside appropriate complementary techniques, are given in Fig. 7. The examples given are far from exhaustive but are provided to highlight the broad applicability of the continuous-flow approach and demonstrate possible auxiliary instrument configurations.

In summary, with regards to the ongoing decline in the overall number of neutron scattering facilities worldwide and the resulting reduction in availability of experimental time, it is clearly crucial for the soft matter community to devise more efficient experiments and maximise the informational content and sample throughput of their neutron scattering experiments. In this regard, the recent and forthcoming improvements in flux and *Q*-ranges at many beamlines represent a major opportunity; as the acquisition times come down, the number of sample systems that are amenable to continuous-flow SANS measurements will increase. This, in turn, will allow users to branch out from the ubiquitous ‘static’ measurements into a wide variety of *in-situ* measurements that have hitherto been performed either predominantly or exclusively with X-rays including: polymerisations^9,75^, nanoparticle synthesis^76,77^ and self-assembly^78,79^ and the characterisation of biological structures^80^. The work presented herein has demonstrated the feasibility and advantages of this approach and lays the foundations for future, more complex sample environment architectures.

## Methods

For each measurement, a 100 mL, 3-necked round-bottomed flask was charged with 50 g of D_2_O and ~0.51 g of oligo-oxyethylene(5) lauryl ether carboxylic acid surfactant (1 wt%, commercially known as AKYPO RLM 45 CA, Kao Corp., Japan). The solution was stirred and continuously circulated through a 1 mm path length flow-cell (Hellma, Germany) positioned in the neutron beam, using a peristaltic pump (100 series, Williamson, UK) at a flow rate of approximately 60 mL/min. Syringe pumps (NE-1010, New Era Pump Systems, USA) were used to dispense NaOD and DCl solutions (0.1 M in D_2_O for the initial measurements and 1 M in D_2_O for the hysteresis measurements). The syringe pumps were controlled remotely throughout the experiment via the ILL instrument control platform, NOMAD. The pD was measured by recording the potential difference over a pH electrode (via the analogue output port of an F20 pH meter, Mettler Toledo, USA) and subsequently converting the result into a pD value by means of a previously performed calibration, noting that pD = pH + 0.4, where pH is the value displayed on the pH-meter^81^. Neutron scattering measurements were carried out at the D11 beamline at the ILL (Grenoble, France). To fully capture the structure of the micelles, three sample-to-detector distances were used (1.5 m, 8 m and 34 m, with collimation lengths of 1.5 m, 8 m and 34 m respectively) with a wavelength of 6 Å, giving a *Q*-range of 0.0015 – 0.42 Å^−1^. Three separate potentiometric titrations were performed. The first consisted of a rapid addition (1.2 mL over 1.2 s, i.e. 60 mL/min) of 1 M NaOD to the aqueous surfactant solution at a detector distance of 8 m (*Q*-range: 0.008 – 0.08 Å^−1^) and measurements were performed continuously with a frequency of 1 s^−1^. The second experiment consisted of a slow addition (12 mL over 2 hours, i.e. 0.1 mL/min) of 0.1 M NaOD to the aqueous surfactant solution. SANS was measured continuously (120 × 60 s measurements) and the procedure was repeated under identical conditions at the three detector configurations. The final experiment, to observe the hysteresis behaviour and effect of salt concentration, consisted of two successive additions of 1 M DCl and 1 M NaOD (1.2 mL over 24 mins, i.e. 0.05 mL/min, paused at 0.6 mL to allow for measurement at additional detector distances). In this case, SANS was measured continuously during addition at a detector distance of 8 m, and additionally at the 1.5 m and 34 m configurations at the beginning, end and midway through each addition. Details of the data reduction are given in the section S1 Supporting Information.

### Data availability

The datasets generated during the current study are available in the ILL data repository^82^: http://doi.ill.fr/10.5291/ILL-DATA.INTER-354.

## Electronic supplementary material

Supplementary Information
