# Supplementary material for: A Small-Angle Neutron Scattering Environment for In-Situ Observation of Chemical Processes
Source: Sci Rep. 2018 May 8;8:7299. doi: 10.1038/s41598-018-24718-z (PMC5940810; doi:10.1038/s41598-018-24718-z)
Supplement: Supplementary file 1 — Supplementary Information [file 41598_2018_24718_MOESM1_ESM.pdf]

A Small-Angle Neutron Scattering Environment for *In-Situ* Observation of Chemical Processes

Dominic W. Hayward[1,2,*], Leonardo Chiappisi[1,2], Sylvain Prévost[2], Ralf Schweins[2], and Michael Gradzielski[1,*]

[1]Stranski-Laboratorium für Physikalische und Theoretische Chemie, Institut für Chemie, Technische Universität Berlin, Straße des 17. Juni 124, D-10623 Berlin, Germany
[2]Institut Laue-Langevin, 71 avenue des Martyrs, CS 20156, 38042 Grenoble cedex 9, France
[*]hayward@ill.fr, michael.gradzielski@tu-berlin.de

S1 Data Reduction

Data reduction was carried out using the ILL software package *LAMP*. Absolute intensities were obtained with reference to the secondary calibration standard, H_2O (1 mm path length), with a differential scattering cross-section of 0.983 cm^{-1} on the D11 instrument with a wavelength of 6 Å. For each measurement, the 2D data were regrouped via integration over the azimuthal angle (0-2π) to produce 1D curves and the solvent background of pure D_2O (in the flow-through cuvette used for all measurements) was subtracted. 1D scattering curves for the same amount of added titrant, taken at different detector distances, were combined and rebinned according to the procedure outlined in section S2. Finally, the data were fitted to the model described in section S3, in absolute units, using the scattering length densities and specific volumes reported previously[1] and provided here in table S1.

Table S1. Summary of the densities, neutron scattering length densities and volumes used in the scattering model.

	Density (g/cm^3)	Neutron SLD (10^{-6} Å^{-2})	Volume (Å^3)
D_2O	1.11	6.39	30
Hydrophobic segment	0.75	-0.31	340
Hydrophilic segment	1.25	1.33	341

S2 Rebinning

In order to simplify the fitting procedure, the data taken from the three instrument configurations were combined into one dataset and rebinned to reduce the number of data points. This was done in three steps:

1. The entire Q-range is split up into N bins distributed evenly in log(Q)
2. Each data point is assigned to its corresponding bin
3. The average intensity and uncertainty is calculated for each bin

S3 Explanation of Model Used to Fit SANS Data

The model used to fit the SANS data has been reported elsewhere[1–3] and is included here for completeness. As outlined in the main article, the self-assembled surfactant is described in two parts: a population of ellipsoidal micelles and population of spherical vesicles. In both cases, core-shell structures are considered where the alkyl chains are assumed to form a homogeneous, anhydrous core (or central shell in the case of vesicles) and the ethylene oxide units, along with the carboxylic acid head group and hydration water (D_2O), form the shell (inner + outer shells in the case of vesicles). The scattering length density of the latter is calculated as the volume-weighted average of the three constituents. Dimensions are shown graphically in Figure S1. In general, the Q-resolution of the instrument should also be considered when fitting experimental SANS data. This can be accomplished either by de-smearing the experimental data or by applying a convolution smearing integral to the fitting function. In this case however, the instrumental resolution was neglected, as the scattering data generally did not show any pronounced features. If the instrumental resolution were considered, although the values of the polydispersity would likely be reduced compared to the observed values, the overall observed sizes of the scatterers would not be affected.

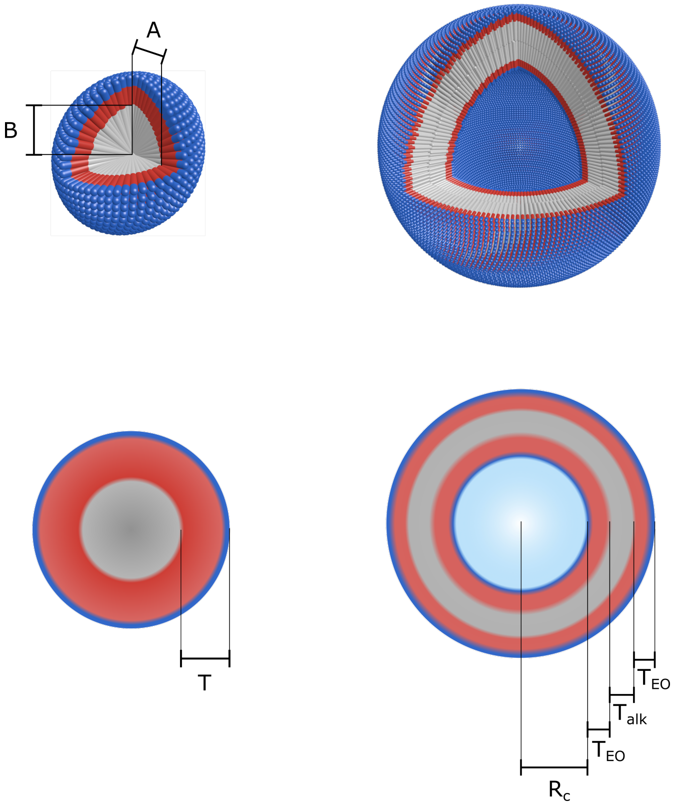

Figure S1. Graphical representation of the dimensions used to describe the micelle and vesicle in the SANS model.

S3.1 Ellipsoidal Micelles

The scattering from the ellipsoidal micelles is calculated using a form factor for core-shell ellipsoids[4] and the structure factor for charged hard spheres where both the hard sphere and electrostatic repulsion are treated in the random phase approximation[5]. The particle number density N is calculated from the ratio of the volume of the micellar core V_{core} to the volume fraction of the alkyl chain ϕ_{core}:

$$N = \frac{\phi_{core}}{V_{core}} = \frac{3\phi_{core}}{4\pi AB^2} \tag{S1}$$

where A and B and the semi-minor and semi-major axes of the ellipsoids respectively. The total scattering intensity is then given by the product of the number density, form factor and structure factor:

$$I_{micelle}(Q) = N \cdot P(Q) \cdot S(Q) \tag{S2}$$

S3.1.1 Form Factor

The orientationally averaged form factor of a core-shell ellipsoidal particle is given by:

$$P_{micelle}(Q) = \int_0^1 |F(Q, \cos\alpha)|^2 \, \mathrm{d}\cos\alpha \tag{S3}$$

where α is the angle between the scattering vector $\mathbf{Q}$ and the semi-minor axis of the ellipsoid. The scattering amplitude is given by:

$$F(Q, \cos\alpha) = (\rho_{core} - \rho_{shell})V_{core}\left[\frac{3j_1(x_{core})}{x_{core}}\right] + (\rho_{shell} - \rho_{solvent})V_{total}\left[\frac{3j_1(x_{total})}{x_{total}}\right] \tag{S4}$$

where ρ_{core}, ρ_{shell} and $\rho_{solvent}$ are the scattering length densities of the core, shell and solvent respectively, j_1 is the first order spherical Bessel function:

$$j_1(x) = \frac{\sin(x) - x\cos(x)}{x^2} \ , \tag{S5}$$

x_{core} and x_{total} are given by:

$$x_{core} = Q\sqrt{A^2\cos^2\alpha + B^2(1-\cos^2\alpha)} \tag{S6}$$

$$x_{total} = Q\sqrt{(A+T)^2\cos^2\alpha + (B+T)^2(1-\cos^2\alpha)}\ , \tag{S7}$$

the volume of the particle core and total volume are:

$$V_{core} = \frac{4}{3}\pi AB^2 \tag{S8}$$

$$V_{total} = \frac{4}{3}\pi(A+T)(B+T)^2\ , \tag{S9}$$

where T is the thickness of the shell. Finally, the water content of the shell, as used in figure Xx in the main text, is calculated from the volume of the shell and the volume of the head group as follows:

$$\phi_{water} = \frac{V_{shell} - N_{agg}v_{head}}{V_{shell}} \tag{S10}$$

where v_{surf} is the average volume of the surfactant headgroup, V_{shell} is the volume of the hydrated shell ($V_{shell} = V_{total} - V_{core}$), and N_{agg} is the aggregation number given by the ratio of the core volume and the volume of the surfactant tails (v_{tail}):

$$N_{agg} = \frac{V_{core}}{v_{tail}} \tag{S11}$$

S3.1.2 Structure Factor

A charged hard sphere structure factor treated in the random phase approximation, after Baba-Ahmed *et al.*[5], was applied to the ellipsoidal micelles to account for the excluded volume and electrostatic interactions between particles. The structure factor takes the general form:

$$S(Q) = \left[1 - N\left(C_0(Q) - \frac{U(Q)}{k_BT}\right)\right] \tag{S12}$$

where k_B is the Boltzmann constant, T is the temperature in kelvin, $C_0(Q)$ determines the hard sphere repulsion and $U(Q)$, the charge perturbation. The hard sphere repulsion is governed by:

$$NC_0(Q) = \frac{A}{x^3}(\sin x - x\cos x) + \frac{B}{x^3}\left[\left(\frac{2}{x^2}-1\right)x\cos x + 2\sin x - \frac{2}{x}\right] - \frac{A\phi_{HS}}{2x^3}\left[\frac{24}{x^3} + 4\left(1-\frac{6}{x^2}\right)\sin x - \left(1 - \frac{12}{x^2} + \frac{24}{x^4}\right)x\cos x\right] \tag{S13}$$

where:

$$x = 2QR_{HS}\ , \tag{S14}$$

$$A = -24\phi_{HS}\left(\frac{1+2\phi_{HS}}{(1-\phi_{HS})^2}\right)^2 , \tag{S15}$$

$$B = -36\left(\phi_{HS}\frac{2+\phi_{HS}}{(1-\phi_{HS})^2}\right)^2 , \tag{S16}$$

ϕ_{HS} is the volume fraction of surfactant and R_{HS} is the hard sphere radius. The perturbation that arises due to the charged headgroups is taken into account with the inclusion of the DLVO interaction potential:

$$\frac{NU(Q)}{k_BT} = \frac{4e_0^2N}{k_BT\varepsilon_0\varepsilon_r}\left(\frac{ZR_{HS}}{2+s}\right)^2\frac{x\cos x + s\sin x}{x(x^2+s^2)} \tag{S17}$$

where $s = 2\kappa R_{HS}$, κ is the reciprocal of the Debye length:

$$\kappa = \sqrt{\frac{2N_AIe_0^2}{\varepsilon_0\varepsilon_rk_BT}}\ , \tag{S18}$$

N_A is the Avogadro constant, e_0, the elementary charge, ε_0, the vacuum permittivity, ε_r, the relative permittivity of the solvent, Z, the effective number of charges per micelle and I, the ionic strength given by:

$$I = \frac{1}{2}\sum_i C_i z_i^2 \tag{S19}$$

where C_i and z_i are the concentration and valency of ionic species i.

S3.2 Vesicles

The scattering from vesicles was described using a polydisperse core-shell sphere model[6]:

$$P_{vesicle}(Q) = \left[\sum_{i=0}^{3} \frac{4}{3}\pi R_i^3 \cdot \Delta\rho_i \cdot 3\frac{\sin(QR_i) - QR_i\cos(QR_i)}{(QR_i)^3}\right]^2 \tag{S20}$$

with:

i	R_i	$\Delta\rho_i$
0	R_{core}	$\rho_{solvent} - \rho_{EO}$
1	$R_{core} + T_{EO}$	$\rho_{EO} - \rho_{alk}$
2	$R_{core} + T_{EO} + T_{alk}$	$\rho_{alk} - \rho_{EO}$
3	$R_{core} + 2T_{EO} + T_{alk}$	$\rho_{EO} - \rho_{solvent}$

where: R_{core}, T_{EO} and T_{alk} are the radius of the inner core, the thickness of the hydrophilic shells and the thickness of the hydrophobic shell, respectively. It is worth noting that the SLD of the hydrophilic shells, ρ_{EO}, also depends on the water content of the shell and therefore the shell thickness:

$$\rho_{EO} = WCS \cdot \rho_{D_2O} + (1 - WCS) \cdot \rho_{dryEO} \tag{S21}$$

where the WCS is the water content of the hydrophilic shell and is given by:

$$WCS = \frac{V_{EO} - V_{alk}\left(\frac{v_{EO}}{v_{alk}}\right)}{V_{EO}} \tag{S22}$$

v_{EO} and v_{alk} are the molecular volumes given in table S1 and V_{EO} and V_{alk} are the combined volume of the inner and outer shell and the volume of the middle shell respectively. These can be calculated by straightforward geometric considerations:

$$V_{alk} = \frac{4}{3}\pi\left[T_{alk}^3 + 3T_{alk}^2(T_{EO} + R_{core}) + T_{alk}(3T_{EO}^2 + 6R_{core}T_{EO} + 3R_{core}^2)\right] \tag{S23}$$

$$V_{EO} = \frac{4}{3}\pi\left[8T_{EO}^3 + 3T_{EO}^2(3T_{alk} + 4R) + 3T_{EO}\left(T_{alk}^2 + 2RT_{alk} + 2R^2\right)\right] \tag{S24}$$

Note that the ratio of molecular volumes, v_{EO}/v_{alk}, is a constant as the hydrophobic and hydrophilic components form a single molecule. In order to capture the polydispersity of the vesicles, a lognormal distribution was applied to the radius of the inner core with a probability density function given by:

$$\mathrm{PDF}(R_{core}, S, M) = \frac{1}{R_{core}S\sqrt{2\pi}}\exp\left[-\frac{(\ln(R_{core}) - M)^2}{2S^2}\right] \tag{S25}$$

with nth moment and variance given by:

$$\langle R_{core}^n\rangle = M^n e^{\frac{1}{2}n^2S^2} \tag{S26}$$

$$\sigma^2 = M^2 e^{S^2}\left(e^{S^2} - 1\right) \tag{S27}$$

The particle number density is calculated from the ratio of the volume of the hydrophobic shell V_{alk} to the volume fraction of the alkyl chain ϕ_{alk}:

$$N(R_{core}) = \frac{\phi_{alk}}{V_{alk}(R_{core})} \tag{S28}$$

where:

$$V_{alk}(R_{core}) = \frac{4}{3}\pi \left[T_{alk}^3 + 3T_{alk}^2(T_{EO} + \langle R_{core}\rangle) + T_{alk}(T_{EO}^2 + 6\langle R_{core}\rangle T_{EO} + 3\langle R_{core}^2\rangle)\right] \tag{S29}$$

As the size of the vesicles is sufficiently large (corresponding to a low number density at the weight fraction used), the structure factor may safely be neglected. The scattering from vesicles can therefore be summarised as:

$$I_{vesicle}(Q) = \int_0^\infty N(R_{core}) \cdot \mathrm{PDF}(R_{core}, S, M) \cdot P_{vesicle}(Q, R_{core})\, \mathrm{d}R_{core} \tag{S30}$$

S3.3 Combined Model

Finally, as the micelles and vesicles co-exist, a further parameter is required to partition the volume fraction into two components, denoted in the main body as 'the fraction of surfactant in micellar form', such that overall function used for fitting the data is given by:

$$I_{total}(Q) = f \cdot I_{micelle}(Q) + (1-f) \cdot I_{vesicle}(Q) \tag{S31}$$

The model was implemented, in the form outlined above, in a *Python* script and the fitting was carried out using the *lmfit* package via the non-linear least squares method.

S4 Measurement and Effect of Flow Rate

The volume flow rate of the pump was established by stopping the pump (with a fully-loaded flow loop), opening the loop and measuring the volume of sample transferred over 60 s. The time taken for the surfactant solution to complete one circulation of the flow loop was determined in a similar manner, by measuring the time taken for the flow loop to empty once the sample outflow tube had been withdrawn from the sample reservoir.

In order to determine the effects of flow (i.e. the continuous pumping of reaction mixture through the observation cell) on the self-assembled structures, SANS measurements were performed on the surfactant solution under both static and flow conditions (with a flow-rate of 60 mL/min, corresponding to a wall shear rate in the observation cell of 615 s^{-1}, at pD 3.2, prior to the addition of NaOH). The scattering patterns and radially averaged intensity data, shown in figure S2a-b, reveal that the scattering is isotropic under static conditions and becomes distinctly anisotropic under flow. As the azimuthally averaged data, shown in figure S2d, do not exhibit any significant differences between the static and flow conditions, this suggests that the morphology of the structures does not change under flow. This, in turn, implies that the morphology of the self-assembled structures is intrinsically anisotropic and the shear forces merely impart a preferred orientation. Based on these observations, the flow-rate was maintained at 60 mL/min for the remainder of the experiment. In order to observe how the degree of anisotropy changes with respect to pD, the data from the single addition of NaOD, observed at a sample-to-detector distance of 8 m ('mid-Q'), over the Q-range 0.02-0.05 Å^{-1}, were analysed using the *SASET* program. The results, shown in figure S3, indicate that the anisotropy decreases to zero in the pD range 3-5, approximately concurrently with the decrease in micelle aspect ratio. This gives further support to the interpretation of initially disc-like micelles becoming more spherical as the pD of the solution increases.

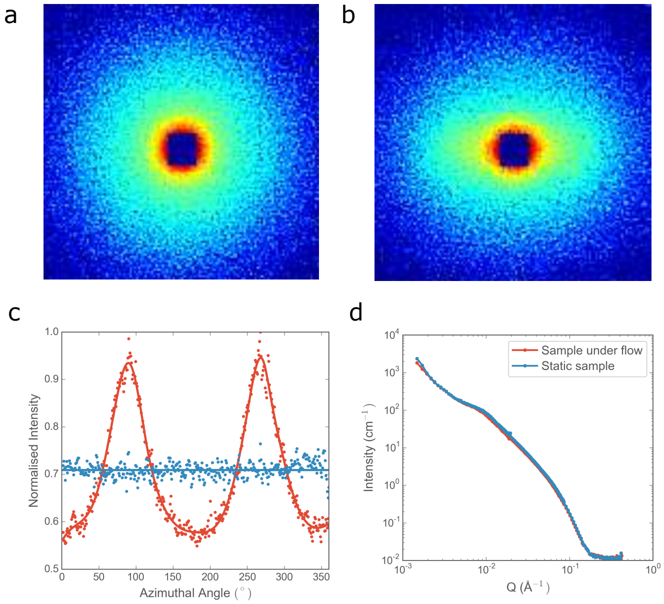

Figure S2. Two-dimensional scattering patterns of surfactant solution at pD 2.9 (a) under static conditions and (b) under flow (flow rate 60 mL/min in vertical direction). The one-dimensional plots show the data from (a) and (b) integrated (c) radially and (d) azimuthally.

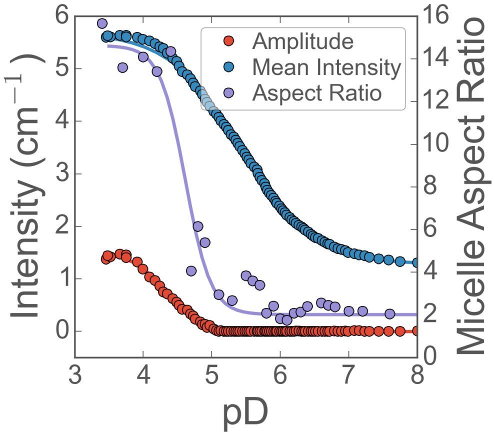

Figure S3. Evolution of the mean intensity and the amplitude of the anisotropy (as fitted with a Gaussian function) for the single addition of NaOD observed under flow (flow rate 60 mL/min in vertical direction) at a sample-to-detector distance of 8 m. Also shown is the corresponding micelle aspect ration calculated from the fitted data shown in Figure 5 of the main text.

S5 Supplementary Figures

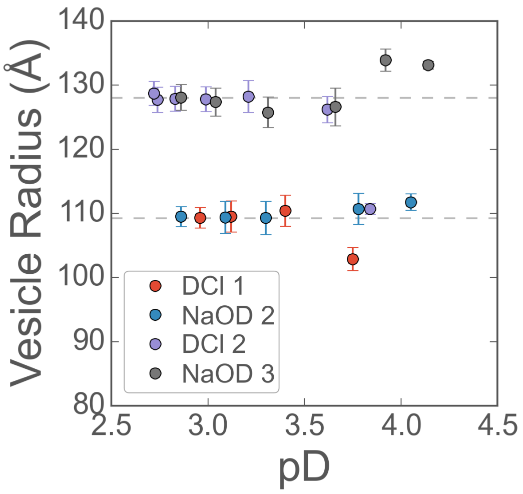

Figure S4. Radius of the vesicles at low pD following sequential additions of NaOD and DCl. The dashed lines represent the mean vesicle radii of 109 Å at an NaCl concentration of 24 mM and 128 Å at an NaCl concentration of 48 mM.

References

1. Chiappisi, L. Polyoxyethylene alkylether carboxylic acids: an overview of a neglected class of surfactants with multiresponsive properties. *Adv. Colloid Interface Sci.* (2017).
2. Chiappisi, L., Prévost, S., Grillo, I. & Gradzielski, M. From Crab Shells to Smart Systems: Chitosan–Alkylethoxy Carboxylate Complexes. *Langmuir* **30**, 10608–10616 (2014). URL http://pubs.acs.org/doi/abs/10.1021/la502569phttp://pubs.acs.org/doi/10.1021/la502569p. DOI 10.1021/la502569p.
3. Chiappisi, L., Prévost, S., Grillo, I. & Gradzielski, M. Chitosan/alkylethoxy carboxylates: A surprising variety of structures. *Langmuir* **30**, 1778–1787 (2014). DOI 10.1021/la404718e.
4. Bendedouch, D. & Chen, S. H. Effect of an attractive potential on the interparticle structure of ionic micelles at high salt concentration. *The J. Phys. Chem.* **88**, 648–652 (1984). URL http://dx.doi.org/10.1021/j150648a003. DOI 10.1021/j150648a003.
5. Baba-ahmed, L., Benmouna, M. & Grimson, M. J. Elastic Scattering from Charged Colloidal Dispersions. *Phys. Chem. Liq.* **16**, 235–238 (1987). URL http://www.tandfonline.com/doi/abs/10.1080/00319108708078524. DOI 10.1080/00319108708078524.
6. Pedersen, J. S. *Neutron, X-rays and Light. Scattering Methods Applied to Soft Condensed Matter* (Elsevier Science, 2002).
